# Quality of pork after electron-beam irradiation: A meta-analysis study

**DOI:** 10.14202/vetworld.2024.59-71

**Published:** 2024-01-08

**Authors:** Teguh Wahyono, Tri Ujilestari, Mohammad Miftakhus Sholikin, Muhlisin Muhlisin, Muhammad Cahyadi, Slamet Diah Volkandari, Endy Triyannanto

**Affiliations:** 1 Research Center for Food Technology and Processing, National Research and Innovation Agency of Indonesia, Gunungkidul 55861, Indonesia; 2 Research Center for Animal Husbandry, National Research and Innovation Agency of Indonesia, Bogor 16911, Indonesia; 3 Faculty of Animal Science, Universitas Gadjah Mada, Sleman 55281, Indonesia; 4 Faculty of Animal Science, Universitas Sebelas Maret, Surakarta 57126, Indonesia

**Keywords:** electron beam, irradiation, meat, meta-analysis, pork

## Abstract

**Background and Aim:**

Irradiation has become a preferred method for pork preservation in recent years. Electron-beam irradiation is notably recognized for its feasibility and safety among various irradiation methods. This meta-analysis study aims to elucidate the impact of electron-beam irradiation on oxidation parameters, color, sensory attributes, and microbiological conditions in pork.

**Materials and Methods:**

A total of 79 data from 22 articles were aggregated into an extensive database. The irradiation dose ranged from 0 to 20 kGy in this current meta-analysis. The observed parameters encompassed oxidation, color, sensory attributes, and microbiological conditions. A mixed-model approach was used to perform the meta-data analysis, in which irradiation dose was treated as fixed effects and distinct studies (articles) as random effects.

**Results:**

Electron-beam irradiation resulted in an increase in thiobarbituric acid-reactive substances levels and peroxide-oxygen value of pork (p < 0.01). Conversely, total volatile-base-nitrogen values (p < 0.05) were observed. Following irradiation, the pH value, lightness (L*), redness (a*), and yellowness (b*) remained unaffected. Pork color tended to decrease after irradiation treatment (p = 0.095 and p = 0.079, respectively) at 7 and 14 days of storage. The irradiation process resulted in an increase in the values of texture and juiciness parameters (p < 0.05). However, electron-beam irradiation resulted in decreased overall acceptability (p = 0.089). In terms of microbiological status, electron-beam irradiation led to a reduction in the populations of *Salmonella* (p < 0.01), *Escherichia coli* (p < 0.01), *Listeria monocytogenes* (p < 0.05), and coliforms (p < 0.05) at 7 and 14 days of storage.

**Conclusion:**

Electron-beam irradiation enhances lipid peroxidation in porcine meat. The color of the meat remained unchanged after treatment. However, with regard to sensory properties, electron-beam irradiation showed a tendency to decreased overall acceptability. Most microbiological parameters decreased following electron-beam irradiation.

## Introduction

Pork is one of the world’s essential sources of muscle food. Pork is a primary meat product in the world and is the most widely consumed food in China, which has the largest population in the world [1]. Pork is one of the world’s most widely consumed foods. In 2023, it is pegged that global pig meat production will reach 123.1 million tonnes, an increase of 0.7% compared to 2022 [2]. Approximately 56% of the world’s pork is produced in Asia (48% in China) (112.5 million tons in 2018). However, Asian pork production has declined significantly since 2019 due to African swine fever [3]. As a result of the high demand for pork, this commodity is the basis for exports to many countries. In 2022, 11.3 million tons of pig meat were exported worldwide, a decrease of 11.3% compared to the previous year. This decrease is primarily attributable to a nearly 45.0% decrease in sales by China [2]. We predict an increase in export mobility in response to the increasing demand after the COVID-19 pandemic. Meat preservation technology is directly related to the export industry.

Methods of meat preservation have been classified into three broad categories based on control by temperature, by moisture, and, more directly, by inhibitory processes (ionizing radiation, packaging, etc.), although specific ways of preservation may involve several antimicrobial basic concepts [4]. Non-thermal preservation may work as the first line of defense against spoilage and pathogenic microflora [5, 6]. In addition to conventional meat preservation methods, ionizing radiation applications have been extensively investigated [7]. Food processing with ionizing radiation offers a wide range of beneficial effects that cannot be obtained by other conventional methods [8]. Current research shows that irradiation reduces microbial contamination, improves shelf life, and preserves nutritional value. However, sensory characteristics may be negatively impacted, requiring additional research [9]. Food irradiation has been widely accepted as an acceptable replacement to chemical preservatives for the treatment of fresh and preserved food products [10]. Electron-beam irradiation is widely used to increase the shelf-life of meat-based products [11] because it is safer than gamma irradiation, it is well known that electron-beam irradiation is more effective than gamma-ray irradiation at reducing *Bacillus cereus* and *Escherichia coli* O157:H7, but not *Listeria monocytogenes* [12]. Electron-beam-accelerators elevate electrons up to 10 MeV and penetrate small objects (5–10 cm) with minimal penetration power [13].

There is an urgent need to conduct a quantitative evaluation due to different results in terms of meat quality, particularly, sensory and quality after irradiation treatment. Several meta-analytical studies have investigated the influence of irradiation on chicken meat quality [14–16]. Furthermore, Fallah *et al*. [17] performed a meta-analysis study on the combination of irradiation and bioactive compounds in muscle food.

To the best of our knowledge, there have been no meta-analyses on the application of electron-beam irradiation on pork. Therefore, the aim of this meta-analysis was to evaluate the effect of electron-beam irradiation on oxidation parameters, color, sensory parameters, and microbiological status in pork meat.

## Materials and Methods

### Ethical approval

This study followed Preferred Reporting Items for Systematic Review and Meta-Analysis guidelines [18].

### Study period and location

The study was conducted from May 12, 2023, to August 21, 2023, at the Research Center for Food Technology and Processing, National Research and Innovation Agency of Indonesia, Indonesia.

### Search strategy

We constructed a comprehensive database based on studies describing the influence of electron-beam irradiation on the oxidation parameters, color, sensory, and microbiological statuses of porcine liver. We conducted a time search for published articles between 1990 and 2023 in Scopus, PubMed, and Google Scholar using the terms “electron beam,” “irradiation,” and “pork.”

### Selection criteria

The following criterion for literature selection was as follows: (1) published in peer-reviewed journals; (2) experiments were performed using electron-beam irradiation instead of gamma irradiation; (3) irradiation dosage was reported; and (4) raw meat or its processed products could be used as the sample.

### Inclusion and exclusion criteria

**Figure-1:**
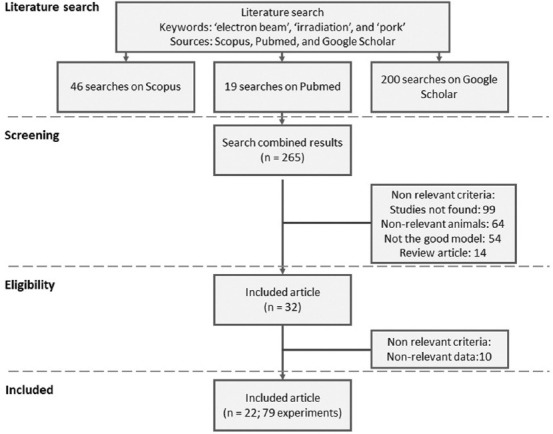
Flow chart for article selection included in meta-analysis.

Figure-1 presents details regarding the selection of studies included in this meta-analysis. The final database consisted of 22 articles with 79 data (Table-1) after reviewing the title, abstract, and document [19–40]. The energy variations in the electron beam in each experiment consist of high energy (10 MeV, 10 studies) and medium energy (0.2–2.5 MeV). However, no information was available in four studies. The irradiation dose ranged from 0 to 20 kGy in this meta-analysis.

**Table-1:** Studies included in the meta-analysis of the influence of electron-beam irradiation on quality of pork.

Study no.	Reference	Meat sample	Energy (MeV)	Dosage (kGy)	Additional treatment
1	Ahn *et al*. [30]	Pork patties	10	0; 1.5; 3; 4.5; 5; and 7.5	Aerobic or vacuum packaging
2	Ahn *et al*. [20]	Pork patties	10	0; and 4.5	Aerobic or vacuum packaging
3	Liu *et al.* [34]	Lean fresh pork	10	0; and 3	0.5% (w/v) D-sodium erythorbate solution; grape seed extract solution; or tea polyphenols solution
4	Ham *et al*. [32]	Pork sausages	10	0; 2.5; 5; 7.5; and 10	-
5	Kim *et al*. [26]	Pork loins	10	0; and 3	Aerobic or vacuum packaging
6	Kim *et al*. [35]	Pork loins	1.5	0; 1; 2; and 3	2% lactic acid; citric acid; or acetic acid
7	Lei *et al*. [36]	Fresh pork	10	0; 1; 3; 5; 7; and 9	Vacuum packaging
8	Kim *et al*. [23]	Pork patties	2.5	0; and 5	10% NaCl or soy sauce
9	Yim *et al*. [28]	Pork shoulder	-	0; and 2	Vacuum packaging
10	Song *et al*. [37]	Minced pork and pork patties	2.5	0; 5; 10; 15; or 20	-
11	Nam and Ahn [21]	Pork homogenates and pork patties	10	0; and 4.5	Gallate; Tocopherol; Trolox; Sesamol; or Carnosine
12	Ohene-Adjei *et al*. [25]	Ground pork and loin chops	-	0; 1.5; and 1.9	Vacuum packaging
13	Kwon *et al.* [38]	Fresh pork	10	0; and 5	Vacuum packaging; or cooked
14	Zhao *et al*. [31]	Pork chops	-	0; and 1	Vacuum packaging; aerobic packaging; 25% CO_2_:75%N_2_ packaging; 50%CO_2_:50%N_2_ packaging; or 75%CO_2_:25%N_2_ packaging
15	Kang *et al*. [39]	Pork jerky	2.5	0; 0.5; 1; 2; 3; and 4	Aerobic packaging; or aerobic packaging+added leek extract (1%)
16	Shin *et al*. [24]	Raw lean pork; and bologna sausage	2.5	0; 2; 4; 6; 8; and 10	Vacuum packaging
17	García-Márquez *et al*. [33]	Fresh pork loin	10	0; 1; and 2	-
18	Yang *et al*. [19]	Fresh pork loin	0.2	0; 8; and 12	Vacuum packaging
19	Zhu *et al*. [40]	Fresh pork loin	-	0; 1.5; and 2.5	Vacuum packaging
20	Kim *et al*. [22]	Porky jerky	2.5	0; 1; 2; and 4	0.5%; or 1% leek extract
21	Kim *et al*. [27]	Porky jerky	2.5	0; 0.5; 1; 1.5; 2; 3; and 4	0.5% Onion peel extract
22	Abeyrathne and Nam [29]	Pork sausages	10	0; and 4	-

Studies included in our meta-analysis were those reporting the effects of electron-beam irradiation on oxidation parameters, color, sensory parameters, and microbiological status in pork meat. Records from studies using gamma irradiation or other sources other than electron-beam sources were excluded from the study.

Oxidation parameters include thiobarbituric acid-reactive substances (TBARS), peroxide-oxygen value (POV), and total volatile base nitrogen (TVBN). TBARS, POV, and TVBN Lightness (L*), redness (a*), and yellowness (b*) were considered color parameters. Sensory parameters included were texture, juiciness, flavor, color, odor intensity, odor preference, and overall acceptability. Microbiological status included total aerobic bacteria, total plate count, *Salmonella*, *E. coli*, *L. monocytogenes*, as well as coliforms observed parameters were the results of storage at refrigerator temperature (<4°C) on days 0, 3, 7, and 14.

### Statistical analysis

We statistically analyzed the meta-analysis data using a linear mixed model approach [41, 42]. Therefore, different studies were considered random effects, whereas electron-beam irradiation doses in pork were treated as fixed effects. The following mathematical models were used to model these effects:


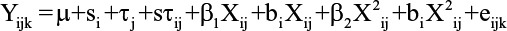


where Y_ijk_ represents the dependent variable, μ be the overall mean value, s_i_ signifies the random effect of the *i*^th^ study, assumed to follow a normal distribution with mean 0 and variance ∼N_iid_ (σ^2^_S_), τ_j_ denotes the fixed effect of the j^th^ τ factor, sτ_ij_ represents the random interaction between the i^th^ and j^th^ levels of the τ factor, also assumed to be normally distributed with mean and variance ∼N_iid_ (σ^2^_Sτ_), β_1_ stands for the overall value of the linear regression coefficient relating Y to X (a fixed effect), β_2_ be the overall coefficient value of the quadratic regression of Y on X (a fixed effect), X_ij_ and X^2^_ij_ correspond to the continuous values of the predictor variable in linear and quadratic forms respectively, b_i_ accounts for the random effect of the study on the regression coefficient of Y to X, assumed to follow a normal distribution with mean 0 and variance *∼N_iid_* (*σ^2^_b_*), and e_ijk_ represents the residual value stemming from unpredictable errors.

The statistical analysis was performed using R software version 4.1.2 developed by R Core Team [43] and the “lme4” package version 1.1–28. Root means square error (RMSE), p-value, and the coefficient of determination (R2) were applied as model statistics. p < 0.05 was considered statistically significant. If p-value was between 0.05 and 0.10, the tendency of 0.10 was considered to indicate statistical significance. We performed all statistical examinations using R version 3.6.3 with the “nlme” library [44].

## Results

### Effect of electron-beam irradiation on oxidation parameters and TVBN in pork

Our meta-analysis findings indicate that electron-beam irradiation increased TBARS value (Table-2) at 0, 3, and 7 d storage (p < 0.01). Regarding oxidation value, electron-beam irradiation increased POV value of pork at 0 and 30 d of storage (p < 0.01). Meanwhile, TVBN value at 0 d storage was decreased after electron-beam irradiation treatment (p < 0.05). Furthermore, TVBN value at 3 d storage also tends to decrease (p = 0.064). TBARS at 14 d and TVBN at 7 and 14 d were not affected by electron-beam irradiation. TBARS at 0 d (mg Malondialdehyde [MDA]/kg) = 0.223 + 0.081X; n = 130; p = 0.002; RMSE = 0.685. TBARS at 3 d (mg MDA/kg) = 0.174 + 0.043X; n = 44; p = 0.004; RMSE = 0.157. TBARS at 7 d (mg MDA/kg) = 0.232 + 0.051X; n = 52; p = 0.003; RMSE = 0.195.

**Table-2:** Influence of electron-beam irradiation on the oxidation parameters and TVBN of pork.

Response parameter	Unit	n	Intercept	SE Intercept	Slope	SE slope	p-value	RMSE	R^2^
TBARS	Mg MDA/kg								
0 day		130	0.22	0.077	0.080	0.017	0.001	0.684	0.01
3 days		44	0.17	0.048	0.042	0.014	0.004	0.157	0.30
7 days		52	0.23	0.055	0.051	0.016	0.003	0.199	0.28
14 days		44	0.43	0.161	0.077	0.051	0.141	0.558	0.18
POV	meq peroxide/kg								
0 day		20	0.64	0.186	0.117	0.035	0.005	0.202	0.73
30 days		20	0.79	0.218	0.129	0.039	0.004	0.218	0.76
TVBN	mg N/100 g sample								
0 day		18	6.06	0.634	−0.188	0.069	0.014	1.874	0.01
3 days		8	8.58	0.128	−0.134	0.047	0.064	0.145	0.65
7 days		8	9.47	0.262	−0.162	0.075	0.118	0.214	0.71
14 days		8	12.0	0.819	−0.503	0.254	0.142	0.747	0.65

TBARS=Thiobarbituric acid reactive substances, POV=Peroxide value, TVBN=Total volatile base nitrogen, SE=Standard error, RMSE=Root mean square error, R^2^=The proportion of a dependent variable’s variation that can be explained by an independent variable (bigger is better)

### Effect of electron-beam irradiation on color and pH in pork

With regard to color parameters, electron-beam irradiation did not affect lightness (L*), redness (a*), and yellowness (b*) of pork, except for a* value at 14 d of storage (Table-3). Furthermore, pH value of pork at 0, 3, 7, and 14 d of storage also not affected by treatment.

**Table-3:** Influence of electron-beam irradiation on color and pH of pork.

Response parameter	Unit	n	Intercept	SE Intercept	Slope	SE slope	p-value	RMSE	R^2^
Lightness (L*)	-								
0 day		72	44.6	3.269	0.107	0.321	0.739	11.08	0.24
3 days		12	52.7	1.508	−0.101	0.198	0.626	0.939	0.85
7 days		34	51.6	1.068	0.127	0.428	0.768	4.595	0.01
14 days		20	51.3	2.376	0.093	0.509	0.858	3.718	0.54
30 days		20	29.4	1.517	−0.238	0.289	0.422	1.671	0.67
Redness (a*)	-								
0 day		72	9.58	0.900	0.029	0.115	0.795	4.008	0.12
3 days		12	8.28	0.804	0.087	0.154	0.589	0.743	0.69
7 days		34	8.76	1.275	0.632	0.512	0.226	5.495	0.01
14 days		20	9.12	1.703	1.514	0.679	0.039	5.677	0.01
30 days		20	8.09	0.806	0.014	0.178	0.939	1.036	0.58
Yellowness (b*)	-								
0 day		72	9.27	0.672	0.024	0.090	0.787	3.168	0.10
3 days		12	9.50	2.417	−0.262	0.751	0.737	3.862	0.31
7 days		34	9.20	0.853	−0.252	0.264	0.348	2.712	0.17
14 days		20	8.92	0.716	−0.084	0.232	0.722	1.812	0.19
30 days		20	12.4	1.423	−0.074	0.329	0.826	1.916	0.55
pH	-								
0 day		32	5.65	0.081	0.037	0.031	0.249	0.269	0.10
3 days		28	5.58	0.086	0.023	0.038	0.547	0.269	0.06
7 days		28	5.63	0.071	0.046	0.034	0.181	0.241	0.01
14 days		24	5.71	0.078	−0.060	0.039	0.144	0.234	0.01

SE=Standard error, RMSE=Root mean square error, R^2^=The proportion of a dependent variable’s variation that can be explained by an independent variable (bigger is better)

### Effect of electron-beam irradiation on sensory parameters in pork

Flavor and odor preference were not affected due to electron-beam irradiation treatment (Table-4). Texture and juiciness parameters decreased as the electron-beam dose increased (p < 0.05). Irradiation increased pork meat odor intensities (p < 0.01). Pork color at 7 and 14 d of storage tends to decrease after treatment (p = 0.095 and p = 0.079, respectively). Electron-beam irradiation tended to decrease overall acceptability (p = 0.089).

**Table-4:** Influence of electron-beam irradiation on sensory parameters of pork.

Response parameter	Unit	N	Intercept	SE Intercept	Slope	SE slope	p-value	RMSE	R^2^
Texture	-	27	6.92	0.564	−0.118	0.042	0.012	0.438	0.88
Juiciness	-	21	6.57	0.462	−0.128	0.045	0.013	0.246	0.93
Flavor	-	27	5.16	0.719	0.095	0.135	0.486	1.456	0.42
Color	-								
0 day		36	6.06	0.284	−0.024	0.036	0.511	0.786	0.29
7 days		16	3.99	1.037	−0.711	0.389	0.095	1.546	0.55
14 days		16	3.19	0.811	−0.638	0.330	0.079	1.331	0.51
Odor intensity	-								
0 days		33	4.47	0.695	0.913	0.138	0.001	1.511	0.67
7 days		8	7.52	2.003	0.233	0.056	0.009	0.211	0.99
14 days		8	7.57	1.489	0.130	0.125	0.345	0.468	0.92
Odor preference	-								
0 day		24	5.9	0.688	0.537	0.137	0.001	1.316	0.58
7 days		16	7.88	0.961	0.031	0.342	0.929	1.758	0.31
14 days		16	6.89	0.627	0.176	0.306	0.575	1.737	0.01
Overall acceptability	-	17	5.24	1.126	−0.146	0.079	0.089	0.428	0.95

SE=Standard error, RMSE=Root mean square error, R^2^=The proportion of a dependent variable’s variation that can be explained by an independent variable (bigger is better)

### Effect of electron-beam irradiation on microbiological status in pork

Most microbiological parameters decreased after electron-beam irradiation (Table-5). Electron-beam irradiation decreased total aerobic bacteria at 0 and 20 d (p < 0.001), compared with the control. Electron-beam irradiation decreased the total plate count on 0 d after storage (p < 0.05). Electron-beam treatment resulted in a decrease in total plate count, *Salmonella*, and *E. coli* populations in pork at 0, 7, and 14 d of storage (p < 0.01).

**Table-5:** Influence of electron-beam irradiation on microbial loads of pork.

Response parameter	Unit	n	Intercept	SE Intercept	Slope	SE slope	p-value	RMSE	R^2^
Total aerobic bacteria	Log CFU/g								
0 day		42	3.01	0.266	−0.236	0.038	0.001	1.232	0.49
30 days		20	4.43	0.264	−1.124	0.079	0.001	0.467	0.92
Total plate count									
0 day		28	2.64	0.372	−2.273	0.578	0.001	1.142	0.00
3 days		28	3.12	0.402	−2.615	0.615	0.001	1.207	0.41
7 days		28	4.36	0.618	−4.160	0.858	0.001	1.638	0.50
14 days		24	5.24	0.766	−4.547	1.158	0.001	1.542	0.51
*Salmonella*									
0 day		38	5.96	0.432	−1.949	0.251	0.001	1.759	0.01
3 days		8	0.15	0.056	−0.064	0.030	0.076	0.082	0.01
7 days		26	4.72	0.814	−1.766	0.531	0.003	2.735	0.01
14 days		22	5.17	0.962	−2.195	0.658	0.003	3.007	0.01
*Escherichia coli*									
0 day		28	5.79	0.653	−1.783	0.333	0.001	2.067	0.01
3 days		12	2.56	1.365	−0.104	0.020	0.001	0.078	1.01
7 days		20	1.18	0.815	0.029	0.368	0.939	2.081	0.05
14 days		12	1.26	0.298	−0.540	0.159	0.007	0.564	0.01
*Listeria monocytogenes*									
0 day		28	5.43	0.628	−1.709	0.320	0.001	1.988	0.01
3 days		8	0.15	0.056	−0.064	0.030	0.076	0.082	0.01
7 days		16	0.58	0.184	−0.250	0.098	0.023	0.411	0.01
14 days		12	1.26	0.298	−0.540	0.159	0.007	0.564	0.01
Coliforms									
3 days		8	0.15	0.056	−0.064	0.030	0.076	0.082	0.01
7 days		16	0.58	0.184	−0.250	0.098	0.023	0.411	0.01
14 days		12	1.26	0.298	−0.540	0.159	0.007	0.564	0.01

CFU=Colony-forming unit, SE=Standard error, RMSE=Root mean square error, R^2^=The proportion of a dependent variable’s variation that can be explained by an independent variable (bigger is better)

*L*. *monocytogenes* populations decreased on 0, 7, and 14 d of storage with increasing irradiation dose (p < 0.05). Electron-beam irradiation decreased coliform population at 7 and 14 d of storage (p < 0.05). Total aerobic bacteria at day 0 (log colony forming unit [CFU]/g) = 3.711 + −0.685X + 0.025X^2^; n = 42; p = 0.001; RMSE = 0.867. Total plate count at day 0 (log CFU/g) = 2.644 + −2.273X + 0.543X^2^; n = 28; p = 0.001; RMSE = 1.142. *Salmonella* at day 0 (log CFU/g) = 6.465 + −3.195X + 0.363X^2^; n = 38; p = 0.001; RMSE = 1.6872. *E. coli* 0 d (log CFU/g) = 7.156 + −4.662X + 0.795X^2^; n = 28; p = 0.001; RMSE = 1.7354. *L. monocytogenes* at day 0 (log CFU/g) = 6.841 + −4.666X + 0.817X^2^; n = 28; p = 0.001; RMSE = 1.619. Coliforms at day 14 (log CFU/g) = 1.71 + −1.89X + 0.45X^2^; n = 12; p = 0.001; RMSE = 0.3403.

## Discussion

This study aimed to determine the impact of electron-beam irradiation on oxidation parameters, color, sensory parameters, and microbiological status in pork. Dimov [14] and Nisar *et al*. [45] identified lipid oxidation by POV and TBARS, respectively. Non-protein nitrogenous substances and the breakdown of proteins produce the TVBN value [46]. Lipid oxidation and protein breakdown affect meat color; therefore, these parameters are critical to meat quality [17]. In addition to nutritional parameters, irradiated meat’s sensory attributes are crucial for the development and marketing of meat-based products [9]. Meat preservation mainly focuses on prevention of microbial spoilage [4]. In the present meta-analysis, total aerobic bacteria, total plate count, *Salmonella*, *E. coli*, *L. monocytogenes*, and coliforms were examined in terms of microbiological status.

### Effect of electron-beam irradiation on oxidation parameters in pork

Electron-beam irradiation generally increases oxidation in pork based on TBARS and POV measurements. The result of the present meta-analysis showed that electron-beam irradiation significantly enhanced the initial TBARS level after 0, 3, and 7 days of storage by p = 0.0001, p = 0037, and p = 0.0025, respectively. To reduce TBARS, a variety of active components and vacuum packaging have been used in most of the experiments submitted for this meta-analysis. However, based on the analysis, this phase of treatment can decrease TBARS value, although it tends to rise after irradiation and storage. TBARS assay is commonly used to measure lipid oxidation in a wide range of muscle foods.

Malondialdehyde, the secondary residue of oxidation of lipid compounds [17, 44], is analyzed in this procedure. Lipid peroxidation reaction generates MDA as a byproduct. This MDA responds with thiobarbituric acid to produce TBARS, a pink chromogen [47]. Ionizing radiation promotes lipid oxidation in muscle foods, leading to deterioration in food quality [17]. When foods are irradiated, many free radicals are generated, which can modify the lipid and protein components of meat [48]. Hydrolysis and oxidation of lipid substances produce aldehydes, ketones, alcohols, and other small molecular substances that directly influence the quality and flavor of meat-based foods [49].

Lipid oxidation, together with microbial spoilage, is a major factor affecting the quality loss of pork products and thus influences the shelf life [49]. Irradiation promotes lipid oxidation, particularly in aerobically packaged meat products, and generates off-odors characteristic of irradiation [50]. Deterioration of meat quality during storage primarily depends on lipid peroxidation and associated alterations [19, 49]. The composition of skin lipids was unchanged significantly, whereas the composition of the polar lipid fractions of muscle was slightly changed [51]. Radiation detection in fat-containing foods is based on the identification of specific lipid molecules generated by irradiation of lipids. 2-Alkylcyclobutanones, for instance, are generated from irradiated fatty acids and triglycerides [52]. The sensitivity of irradiated muscle tissues to lipid oxidation is correlated with the nature, proportion, and level of saturation in fatty acids and the overall structure of phospholipids in the cell membrane [53].

A previous meta-analysis [14] reported that gamma irradiation increased lipid oxidation TBARS levels in chicken meat (p = 0.001). Wahyono *et al*. [16] also reported that electron-beam irradiation enhances TBARS levels in chicken and duck meat. The increased TBARS value level could be different depending on the lipid content and sensory characteristics of the meat source. Various animal species can be categorized according to their meat’s sensitivity to oxidation in the following order: fish > turkey > chicken > pork > beef > lamb [51, 54]. The fat content as well as fatty acid composition of muscle foods are the most influential intrinsic factors on initial lipid oxidation levels [17]. Therefore, the total fat content of pork was a crucial factor in determining its storage stability [20]. The matrix and composition of the irradiated sample appears to affect TBARS [55]. Therefore, antioxidants are likely to be one of the most beneficial treatments for reducing fat oxidation [21–24]. Interestingly, the TBARS value increased slowly when samples were irradiated and combined with antioxidants compared to radiation-treated and untreated samples [11]. Antioxidants can reduce TBARS levels; therefore, a combination treatment is necessary to preserve meat.

The trend of POV increases in pork after gamma irradiation is consistent with the pattern of TBARS value increase. All the studies summarized in present meta-analysis confirmed an increase in POV after irradiation. TBARS and POV are two parameters, one of which represents lipid oxidation, according to the studies, we compiled. In our meta-analysis, TBARS and POV are presented simultaneously. POV is an essential quality factor in irradiated meat samples, indicating the extent of lipid damage induced by irradiation [56]. Hydroxyl radicals (OH) commonly initiate lipid oxidation in muscle tissue by interacting with conjugated systems [57]. Lipid hydroperoxides are the main by-products of lipid oxidation; however, peroxides are eventually transformed [23]. Therefore, measuring the concentrations of peroxides in the pork samples to evaluate the extent of oxidation seems acceptable.

In muscle foods, microbes and/or endogenous proteolytic enzymes degrade proteins and other nitrogenous substances to generate ammonia and organic amines, also known as TVBN [17]. The TVBN index is commonly applied to evaluate the freshness of a variety of meat products [24, 58]. In present meta-analysis, TVBN value at 0 and 3 d of storage was decreased after electron-beam irradiation by p = 0.015 and p = 0.064, respectively. Reduction in TVBN values is likely due to a decrease in the microbial population following radiation. Reducing the number of microorganisms may lead to a reduction in protein degradation in pork. According to a previous meta-analysis [17], irradiation resulted in a 63.4% (R* = 0.366) significant decrease in the TVBN concentration in muscle foods during storage. The TVBN values tend to increase during storage. This mechanism can be inhibited by irradiation treatment.

Chen *et al*. [11] showed that irradiation before storage inhibited increases in TVBN values compared to the untreated sample. Electron-beam irradiation was applied to both superchilled and chilled pork steaks, reducing TVBN levels. Increased dose levels increased the effect [19]. According to Chen *et al*. [11], irradiation influences the TVBN value through two mechanisms: (1) the radiation-induced breakdown of nitrogen-containing substances in meat products enhances the TVBN value; and (2) the irradiation treatment could have limited growth and reproduction of bacteria, which led to a large amount of TB-N.

### Effect of electron-beam irradiation on color and pH in pork

In the present meta-analysis, it is necessary to evaluate color characteristics as a significant indicator of pork market quality. The methods applied to preserve meat primarily focus on preventing microbial spoilage, although all preservation techniques are designed to minimize color and oxidation changes [4]. According to a meta-analysis, electron-beam irradiation did not significantly affect pork color. This is represented by parameters lightness (L*), redness (a*), and yellowness (b*) remaining unchanged. Theoretically, oxidation leads to a mechanism that changes meat color. Irradiation initially resulted in darker (decreased L* value), redder (increased a* value), and yellower (decreased b* value) minced pork [25]. Lipid oxidation may promote myoglobin oxidation. Therefore, the factors that influence lipid oxidation in meat may also affect meat color [53, 57].

Interestingly, our findings are contradictory. This is probably because in most of the experiments, vacuum packaging and antioxidants were applied to the samples. Ahn *et al*. [20] reported that electron-beam irradiation had a significant (p < 0.05) influence on color aspects (L*, a*, and b* values) in pork patties with various packaging-irradiation, but packaging variables of the meat might also be a key aspect.

Regarding L* values, a number of previous investigations have also indicated that these values are unaffected by irradiation. When packaged aerobically, the L* values of turkey and pork were unaffected by irradiation and storage [26]. However, the color values of samples after including leek extracts or onion peel extract were inconsistent during storage [20, 25]. Yim *et al*. [28] demonstrated that inconsistent color changes were observed during aging, temperature, and exposure to irradiation. Low-dose irradiation did not affect the meat color or cause discoloration in pork shoulder at all ages. Increasing irradiation also had no impact on the L* values for pork and turkey [59].

Electron-beam or X-ray irradiation did not affect the color of irradiated pork sausage compared to non-irradiated pork sausage [24]. With or without irradiation, there was no significant change in the L* value of sausage made from hot or cold carcasses during storage [29]. Due to the addition of other ingredients, discoloration does not occur specifically in various meat-based foods. Although irradiation enhances the color of minced meat, it is difficult for processed meat products [60].

Irradiated metmyoglobin (MetMb) creates oxymyoglobin (OxyMb), which increases a* values [15, 56]. The effect of irradiation on the color of meat is dependent on the form of myoglobin. Color changes in irradiated raw meat occur by the myoglobin molecule’s inherent sensitivity to energy input and alterations in the chemical environment, haem iron especially susceptible [4]. On the contrary, our meta-analysis study reported that electron-beam irradiation did not change a* value on 0, 3, 7, and 30 days of storage. The value of a* increased significantly only after 14 days of storage. (p = 0.039). Similar inconsistent results were reported by Luchsinger *et al*. [61], Kim *et al*. [26], and Kim *et al*. [23]. Luchsinger *et al*. [61] demonstrated that the color a* values of aerobically packaged pork chops were not changed on 0 and 3 d of storage, but lowered on 7 d of storage.

The color a* values of pork preserved in vacuum packaging for a week increased slightly regardless of irradiation, but they failed to show a consistent trend when stored in aerobic packaging [26]. Irradiated MetMb generates OxyMb as a red pigment [15]. Pork samples changed pink on irradiation due to the formation of a carbon monoxide-myoglobin complex, caused by carbon monoxide generation and irradiation-decrease conditions [20, 62].

Our meta-analysis study reported that electron-beam irradiation did not change the b* value on 0, 3, 7, 14, and 30 d of storage. Inconsistent results have been reported regarding irradiation-induced changes in b* values [27, 59]. Several variables influence the color of irradiated meat, including heme pigment concentration (particularly myoglobin), oxidation condition, ligand formation, and physical properties (irradiation dosage, temperatures, pH, and storage duration) [9]. Irradiation may alter the myoglobin pigment in meat, resulting in a change in redness (a*). However, according to our meta-analysis, there was no significant change in the level of yellowness in pork (b*) after Irradiation. According to Montiel *et al*. [63], electron-beam irradiation significantly changed L* and a* value of seafood (cold smoked salmon), but had no effect on b* value. Moreover, we suggest that variations in the effect of radiation on protein denaturation and water-holding capacity in pork may account for the inconsistency of b* value results between studies. However, there is a need for further investigation. Variations in muscle structure could also influence meat color without affecting pigment level [64]. Numerous factors, such as irradiation dose, animal species, muscle type, additives, and packaging type, influence the color changes of meat irradiated [65].

In general, in our meta-analysis, the pH value of pork remained unchanged after irradiation. We hypothesize that irradiation inhibits lactic acid synthesis by glycogen hydrolysis [66] and limits the growth and reproduction of pathogens in meat [11]. We hypothesize that these mechanisms are related to the constant pH value. The preservation process, which is the primary objective of irradiation, performs optimally in the presence of a stable pH. pH is a crucial parameter for determining the shelf life of meat products. Electron-beam irradiation tends to preserve the pH level during storage. This will increase the shelf life of pork. In addition to possessing undesirable sensory qualities, meat with short shelf life is influenced by a high pH value, which promotes microbial growth [67].

### Effect of electron-beam irradiation on sensory parameters in pork

Texture is an important factor in the assessment of meat products [68]. The reduced texture and juiciness of irradiated pork is due to oxidation, which influences muscle protein. Changes in texture may be related to protein oxidation following irradiation. Cross-linking or cleavage induced by protein oxidation can alter seafood texture and water-holding capacity [69]. The myofibrillar protein profile of meat decreases with increasing irradiation dose. It affects the secondary structure, which modifies the functional properties of the myofibrillar protein, thus influencing the texture of the meat [9]. Irradiation weakens the texture of the meat, especially at high doses. This might happen as a result of moisture loss caused by drip or purge, which may affect tenderness [25]. High-energy radiolysis of water may generate radiolytic products that can change the qualities of meat, such as water holding capacity and texture [24]. Lipid oxidation has a negative impact on the texture of meat [5, 66] in addition to protein. Indiarto *et al*. [9] reported that irradiation induces the breakdown of connective tissues in meat, resulting in a softer texture. In addition, irradiation has also been shown to be associated with a decrease in the elasticity of cartilage. Texture changes in irradiated meat are influenced by the dose level, pH, packaging method, storage duration, storage temperature, addition of antioxidants, and muscle type [9, 68, 69]. Temperature after irradiation influences the texture of Pork. Freezing could improve the texture of irradiated meat [70]. Moreover, the retention of textural quality caused by irradiation at subfreezing temperatures suggests a freezing versus non-freezing condition, rather than an influence of temperature impact.

Pork’s organoleptic quality can be optimized by balancing flavor and texture [3]. This irradiation may affect the flavor of the meat. The breakdown by products of lipid oxidation, such as aldehydes, alcohols, hydrocarbons, ketones, and furans, may lead to the loss of flavor in irradiated meat as well as meat products [5]. Protein oxidation after irradiation also induces significant modifications in meat, affecting its nutritional, functional, and sensory properties (flavor, texture, color, and juiciness) [67]. In the present meta-analysis, electron-beam irradiation has no significant impact on pork flavor. This condition may be caused by: (1) irradiation technology can significantly eliminate microorganisms in foods under low-temperature conditions to guarantee the safety of food while maintaining its flavor quality [69]; and (2) there is a combination of packaging and additive treatment in the studies investigated to maintain the level of meat flavor [4, 9]. Similarly, Panseri *et al*. [71] reported that irradiation did not affect food ingredients, such as free amino acid pools, thereby preserving the original meat quality. Changes in flavor also depend on the volatile compound content of the additional components of meat products. Oxygen (O_2_) facilitates the initiation of oxidation reactions by volatile chemicals, thereby altering meat flavor characteristics [72].

Similarly, our meta-analysis indicated that electron-beam irradiation had little effect as a sensory parameter on pork color. Color was not affected by electron-beam irradiation on 0 d of storage. However, meat color tended to decrease after treatment (p = 0.095 and p = 0.079, respectively) at 7 and 14 d of storage. Pork quality is primarily determined by its texture, flavor, and color [29]. In addition, the color of meat and meat products mainly comes from the pigments contained in the meat. Susceptibility of the myoglobin molecule, specifically iron, leads to color changes in freshly irradiated meat [73]. Pork is referred to as red meat because it contains more myoglobin compared to poultry and fish. After irradiation, myoglobin’s free binding sites may react with free radicals created by irradiation to generate MetMb, the compound responsible for its brown color [74]. To minimize meat discoloration [75], several packaging and processing techniques for pork have been designed.

Irradiation stimulates lipid peroxidation by generating superoxides and OH, resulting in unfavorable odors, color changes, as well as a decrease in shelf life [52]. Irradiation triggers or enhances lipid oxidation, leading to unwanted odors and flavors [27]. Dimethyl sulfoxide, an attribute off-odor substance in irradiated meat, was detected in materials created by the radiolysis of methionine exposed to 4.5 kGy of irradiation [76]. Radiation treatment has also been shown to affect the oxidation-reduction ability of meat by accelerating lipid oxidation, protein breakdown, as well as flavor and odor change [9]. In contrary, in a special case, Arvanitoyannis and Stratakos [77] demonstrated that irradiation had no impact on the production of volatiles that result from lipid oxidation, but it resulted in a few compounds containing sulfur that were not present in non-irradiated meat.

Most of the irradiation odor was generated by sulfur-containing compounds; however, they volatilized quickly following storage under aerobic environments [30]. Zhao *et al*. [31] reported that a combination of O_2_ and unsaturated fats could accelerate irradiation odor formation. After irradiation, odor is an important factor that determines consumer acceptance. As reported by Ahn *et al*. [30], the odor of irradiation persisted longer in frozen than in refrigerated pork patties, and panelists could identify the odor even after 3 months of frozen storage. There is a significant negative effect on the texture, juiciness, and color variables, and the odor intensity also increases, which tends to reduce the overall acceptability. Even if the taste has not changed significantly, the first impression of the meat has a negative impact on the consumer. Meats’ sensory qualities (texture, flavor, color, and odor intensity) play an important role in evaluating their overall quality and acceptance by consumers [9].

### Effect of electron-beam irradiation on microbiological status in pork

The purpose of using ionizing radiation to preserve pork is to eliminate pathogenic microorganisms present in the meat. The present meta-analysis reported the optimum electron-beam irradiation dose to remove total aerobic bacteria, *Salmonella*, *E. coli*, *L. monocytogenes*, and coliforms to be 13.56, 4.41, 2.93, 2.86, and 2.10 kGy, respectively. As reported by Tahergorabi *et al*. [78], the mechanism to eliminate microorganism contamination from meat generally includes both direct and indirect processes. Microbial inactivation attacks the genetic material (DNA and RNA) and destroys guanine–cytosine and thymine–adenine base pairs, leading to pathogen reproductive death. The indirect effect of e-beam removal of microorganisms is due to the destruction of cell membrane by free radicals. Water radiolysis generates free radicals that degrade microorganism DNA [79]. This “ionizing” effect destroys the DNA, proteins, and cell membranes of bacteria [80] by breaking water molecules into hydrogen, hydroxyl, and O_2_ radicals. Radiation engages with water and the various biological substances in a food system to generate several kinds of radiolytic products, which work as oxidizing agents and may lead to a number of modifications in the molecular components of organic matter [81].

Total aerobic bacteria are an important indicator of microbial contamination because they have significant effects on the shelf life of animal products. Normally, non-irradiated control meat products are spoiled due to an inflated packaging pouch as a sign of growth-induced gas production [32]. Previous studies demonstrated that total aerobic bacteria after irradiation treatment were below the detection limit [25, 43, 79, 80], therefore, the samples were considered contaminant-free. The findings indicate that electron beam is highly effective at removing total aerobic bacteria from meat products. Irradiation, including electron-beam irradiation, has a bactericidal effect because O_2_ and OH destroy the DNA of microorganisms [22]. Therefore, our present meta-analysis showed that electron-beam irradiation succeeded in reducing total aerobic bacteria in pork even after 30 d of storage. Mantilla *et al*. [82] and Shankar *et al*. [83] reported significantly lower bacterial counts of irradiated meat during storage compared to untreated meat. Numerous factors, such as water activity, food ingredients, irradiation or storage temperature, and the presence of O2, affect the D10 values of microbes in food [27]. Most radiosensitive bacteria are efficiently eliminated by irradiation in a mixed microorganism, such as fresh meat [33].

High protein and lipid levels in pork products promote the growth of pathogens that cause foodborne illness [29]. A high concentration of Gram-negative pathogens such as *E. coli*, *Staphylococcus* spp., and *Salmonella* in meat products could substantially also reduce its shelf life and texture quality [84]. *E. coli* is a notable pathogenic pathogen that causes health risks to humans. Controlling these pathogenic bacteria in food is essential for consumer protection [27, 83]. Although Gram-positive and Gram-negative pathogenic bacteria have different levels of radiosensitivity, they are both able to be eradicated by electron-beam irradiation. In contrast to *L. monocytogenes* (Gram-positive), *Salmonella* (Gram-negative) has been shown to be the most susceptible to treatment, demonstrating the greatest resistance. In addition to the radiosensitivity level of pathogenic bacteria, the indirect effect of the production of free radicals has an important effect on their elimination.

It is believed that some complex food system components, such as proteins, compete with cells for associations with radiolytic free radicals, thus decreasing the net effect of damage caused by radiation and making bacteria more radiation-resistant [27]. Zhao *et al*. [31] reported that *Salmonella* was generally more resistant to irradiation when it is irradiated under anaerobic conditions (vacuum or a high level of CO_2_). Moreover, during storage, *Salmonella* continued to grow in unirradiated pork. The number of *Salmonella* survivors did not increase during storage in samples sealed with CO_2_ atmospheres, and no *Salmonella* was detected in vacuum or CO_2_-packed samples after 2 weeks of storage.

## Conclusion

The main purpose of irradiating pork with electron beams is to prevent microorganism proliferation and reproductive ability. However, this present meta-analysis aimed to explore the impact of electron-beam irradiation on oxidation parameters, color, sensory attributes, and microbiological status of pork. The results of this meta-analysis revealed that electron-beam irradiation amplified pork lipid peroxidation. On the other hand, after irradiation, the color of the meat remained unchanged. Nevertheless, electron-beam irradiation resulted in a reduction in both texture and color attributes for sensory characteristics. In addition, irradiation exhibited a propensity to diminish the overall acceptability due to an increase in odor intensity following storage. The current study also implies that irradiation holds the potential to curtail microbial contamination, including total aerobic bacteria, *E. coli*, *Salmonella*, *L. monocytogenes*, and coliforms. Electron beam remains an effective method for pork preservation. Nevertheless, it becomes imperative to integrate this treatment with advanced packaging technology, the incorporation of antioxidants, and precise storage method to alleviate certain unfavorable outcomes.

## Authors’ Contributions

TW, TU, and MMS: Conceptualization and Supervision. TW, MMS, and MC: Methodology and formal analysis. MC, MM, and SDV: Validation. TW, MM, and ET: Investigation. TW, MMS, and SDV: Resources and writing review and editing. TW and MMS: Data curation and writing original draft. TW, TU, MM, and ET: Revised and edited the manuscript. All authors have read, reviewed, and approved the final manuscript.
